# Efficacy and Safety of Open-Source Hybrid Closed-Loop Automated Insulin Delivery in Perioperative Patients

**DOI:** 10.3390/biomedicines14051098

**Published:** 2026-05-13

**Authors:** Delin Ma, Weijie Xu, Yan Yang, Lingyan Bai, Junhui Xie, Jing Tao, Simiao Xu, Kun Dong, Xiaoli Shi, Xiaoqing Song, Yurong Zhu, Nan Sun, Guomin Huang, Fang Liu, Xianlong Hu, Jia Li, Mengran Li, Tangdong Ao, Jingyi Yuan, Xuefeng Yu, Zhelong Liu

**Affiliations:** 1 Division of Endocrinology, Department of Internal Medicine, Tongji Hospital, Tongji Medical College, Huazhong University of Science and Technology, Wuhan 430030, China; dma@hust.edu.cn (D.M.); xwj.07@163.com (W.X.); yangyan@tjh.tjmu.edu.cn (Y.Y.); m202576713@hust.edu.cn (L.B.); junhui_xiexie@sina.com (J.X.); tjskc@126.com (J.T.); tjxusimiao@126.com (S.X.); kundong2009@aliyun.com (K.D.); fll766@163.com (X.S.); song2014xs@163.com (X.S.); zhuyurongdx@163.com (Y.Z.);; 2 Hubei Clinical Medical Research Center for Endocrinology and Metabolic Diseases, Wuhan 430030, China; 3 Branch of National Clinical Research Center for Metabolic Disease, Wuhan 430030, China; 4 Department of Nursing, Tongji Hospital, Tongji Medical College, Huazhong University of Science and Technology, Wuhan 430030, China; 5 Wuhan United Imaging Healthcare Surgical Technology Co., Ltd., Wuhan 430206, China; fang.liu02@uih-surgical.com (F.L.); xianlong.hu@uih-surgical.com (X.H.); jia.li@uih-surgical.com (J.L.); mengran.li@uih-surgical.com (M.L.); tangdong.ao@uih-surgical.com (T.A.); jingyi.yuan@uih-surgical.com (J.Y.)

**Keywords:** automated insulin delivery, open source, hybrid closed loop, type 2 diabetes mellitus, elective surgery

## Abstract

**Background:** Evidence supports the effectiveness and safety of open-source automated insulin delivery (AID) in patients with type 1 diabetes. However, evidence regarding the clinical application of open-source AID in perioperative patients with type 2 diabetes remains limited. **Methods:** This was an open-label, single-center, exploratory pilot randomized controlled trial (RCT) with parallel groups. Patients with diabetes (excluding type 1 diabetes mellitus) scheduled for elective surgery were randomly assigned to the closed-loop group (open-source hybrid closed-loop AID system) or the control group (conventional insulin pump). The primary outcome was the percentage of time in the target glucose range (TIR, 3.9–10.0 mmol/L). Other efficacy and safety outcomes were also compared between the groups. **Results:** A total of 49 participants were included and randomized to the closed-loop group (n = 25) or the control group (n = 24). Participants underwent abdominal, orthopedic, thoracic surgery, or neurosurgery during hospitalization. Patients in the closed-loop group had significantly higher TIR than patients in the control group (76.4 ± 14.1% vs. 61.2 ± 20.0%, *p* = 0.005). Compared with the control group, the closed-loop group also exhibited a 15.6 percentage point reduction in time above range (TAR, >10 mmol/L) without increasing time below range (TBR, <3.9 mmol/L). There were no episodes of severe hypoglycemia (<2.2 mmol/L) or diabetic ketoacidosis in either group. **Conclusions:** This study demonstrates that in patients with diabetes undergoing elective surgery, the open-source hybrid closed-loop AID system provides better glycemic control than conventional insulin pump therapy.

## 1. Introduction

With economic development and population aging, diabetes has become a global public health problem affecting human health. The prevalence of diabetes among adults in China has reached 11.2% [1]. Meanwhile, the proportion of diabetic patients among surgical patients is also gradually increasing, accounting for 10% to 20% [2,3]. Stress factors such as surgery, anesthesia, and psychological tension can further aggravate perioperative hyperglycemia in diabetic patients. The incidence of perioperative hyperglycemia can reach 20% to 40% in general surgery patients, 75% in gastrointestinal surgery patients, and 80% in cardiac surgery patients [4,5,6]. Perioperative hyperglycemia leads to metabolic and functional disorders in the body, aggravates organ damage, and increases the risk of postoperative infection and even death [7]. Therefore, perioperative blood glucose management is of vital importance.

Subcutaneous insulin injection remains the preferred therapy for glycemic management in diabetic patients during the perioperative period. The most frequently employed insulin delivery regimens are multiple daily insulin injections (MDIs) and continuous subcutaneous insulin infusion (CSII), also known as the conventional insulin pump. In comparison with MDI therapy, CSII therapy can better reduce the glycated hemoglobin level of diabetic patients, shorten the time to reach the blood glucose target, and reduce the occurrence of hypoglycemia [8,9]. In surgical patients with diabetes, our earlier studies also confirmed that, compared with non-CSII insulin regimens, CSII therapy achieves better glycemic control and lowers the risk of postoperative infection [10,11].

**Figure A1:**
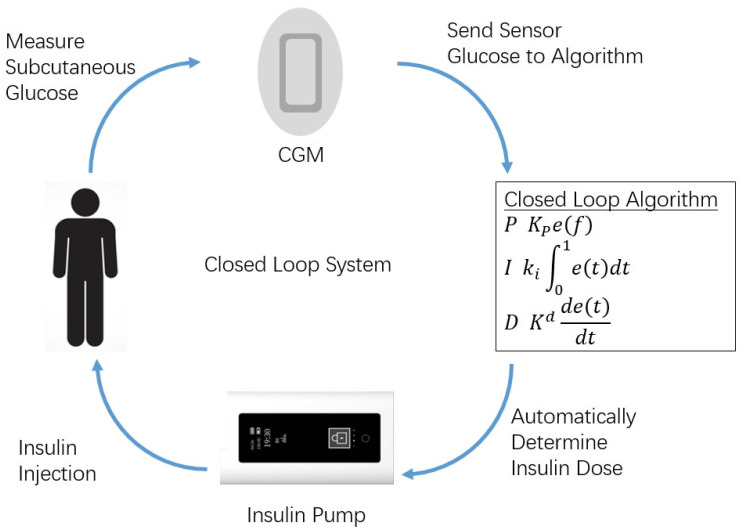
Schematic diagram of an artificial pancreas system.

In recent years, in addition to the traditional MDI and CSII treatments, the artificial pancreas system (Artificial Pancreas, AP) has attracted increasing attention as a novel diabetes treatment option. The artificial pancreas system, also called the automatic insulin delivery (AID) system, mainly consists of three components: a continuous glucose monitor (CGM), an insulin pump, and a control algorithm (Figure A1). The control algorithm can automatically calculate and adjust the insulin infusion rate based on the real-time glucose levels to achieve closed-loop blood glucose management, also known as a closed-loop system [12,13].

At present, there are several closed-loop control algorithms used in AID [14], but these commercial AIDs do not allow for individualized adjustment of insulin infusion parameters. Meanwhile, the open-source AID system, initially developed by the type 1 diabetes patient community, has gradually been applied to diabetic patients [15,16], and studies have proved that both open-source AID and commercial AID are able to achieve satisfactory glycemic outcomes [17,18]. There are mainly three open-source algorithms: OpenAPS, which is compatible with both iOS and Android; AndroidAPS, which targets Android devices exclusively; and Loop, designed for iOS. Despite minor implementation differences, all three share nearly identical design philosophies. Although open-source AID systems are well-established in type 1 diabetes [19,20,21], evidence in type 2 diabetes is limited [22], and their use in perioperative type 2 diabetes patients in China is unreported. This study aims to address this gap by evaluating an open-source hybrid closed-loop system in this setting. The open-source approach, with its inherent advantages of lower cost, greater transparency, and higher customizability, holds particular promise for resource-limited settings.

The aim of the present study is to compare the efficacy and benefits of the hybrid closed-loop artificial pancreas system and the conventional insulin pump combined with CGM in diabetic patients during the perioperative period.

## 2. Materials and Methods

### 2.1. Study Design and Participants

**Figure 1:**
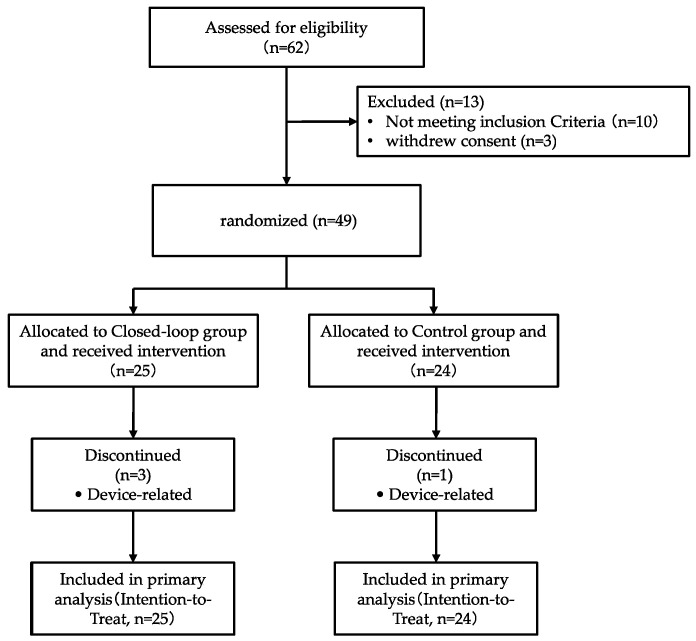
Flow diagram of participant screening, enrollment, randomization, and follow-up in this randomized controlled trial.

This study was an open-label, single-center, exploratory pilot randomized controlled trial (RCT) with parallel groups, designed and reported in accordance with the CONSORT 2025 statement. Participants were recruited from the surgical departments of Tongji Hospital (affiliated to Tongji Medical College, Huazhong University of Science and Technology) from October 2023 to January 2025. Written informed consent was obtained from all participants prior to study initiation. The research protocol was approved by the Ethics Committee of Tongji Hospital (Approval Number: TJ-IRB202308116; Approval Date: 31 August 2023) and registered at ClinicalTrial.gov (NCT06295289). The participant flow diagram is illustrated in Figure 1.

We included patients with type 2 diabetes mellitus (T2DM) or other types of diabetes except type 1 diabetes mellitus (T1DM), aged between 18 and 75 years, who were scheduled for elective surgery and anticipated to have an inpatient hospital stay of more than 72 h. The diagnosis of diabetes was made in accordance with the Chinese Guidelines for Diabetes Prevention and Treatment. Exclusion criteria included (1) acute diabetic emergencies, including diabetic ketoacidosis (DKA) or hyperosmolar hyperglycemic state (HHS); (2) severe cardiac dysfunction (New York Heart Association class ≥ III) or renal impairment (serum creatinine > 442 μmol/L); (3) documented hypersensitivity to protocol-specified medications; (4) standard contraindications to insulin pump therapy; (5) history of allergic diathesis or medical adhesive intolerance; (6) active dermatological diseases or coagulopathies; (7) cognitive impairment or psychiatric conditions that preclude informed consent; (8) investigator-determined unsuitability for trial participation; and (9) occurrence of major perioperative complications. All participants were recruited during the perioperative period in accordance with the above inclusion and exclusion criteria.

### 2.2. Procedures

All participants who met the inclusion/exclusion criteria signed written informed consent forms and were randomly assigned to the open-source hybrid closed-loop automated insulin delivery (AID) group (closed-loop group) or the control group. The random allocation sequence was generated by an independent statistician not involved in patient recruitment or assessment using a computer-generated random number table. Allocation concealment was achieved using sequentially numbered, sealed, opaque envelopes to ensure concealment. Participants of both groups were equipped with identical devices, including Medtronic 712E insulin pumps (Northridge, CA, USA), CGM systems (SiBionics Company^®^, Shenzhen, China), Android smartphones (Redmi OR Huawei, Shenzhen, China), and Ascensia CONTOUR^®^ glucose meters (Basel, Switzerland). Baseline demographic and clinical data, including age, body mass index, diabetes duration, glycated hemoglobin (HbA1c), and surgical type, were recorded at the time of enrollment.

The insulin pump and CGM sensor were implanted by nursing staff on abdominal or upper arm sites, avoiding surgical areas. Insulin aspart (Fiasp; NovoNordisk, Bagsværd, Denmark) was delivered via the pump, and all other glucose-lowering agents were discontinued during the study.

The hybrid closed-loop system, utilizing the open-source artificial pancreas algorithm AMA and operating on the Oref0 control algorithm (OpenAPS Reference Design Zero), required manual input of participant weight and total daily insulin dose for initialization. Glucose targets (6.0–8.0 mmol/L) were individualized based on hypoglycemia risk. The hybrid closed-loop system automated basal insulin delivery, whereas manual input was required for prandial boluses. Real-time glucose levels measured by the CGM were transmitted via the xDrip+ application to the algorithm, which was embedded along with xDrip+ in an Android smartphone. While the control algorithm autonomously regulates the basal insulin delivery, nursing staff are responsible for manually administering meal boluses based on medical orders prior to meals. Dose calculations, incorporating real-time glucose levels, trends, and insulin on board (IOB), were performed at 5 min intervals, and the computed instructions were relayed via Bluetooth to the insulin pump for infusion. The maximum basal rate was limited to three times the preset value to reduce the risk of overdose. Insulin delivery was automatically suspended when glucose levels fell below 4.4 mmol/L.

In the control group, participants received conventional insulin pump therapy with the same devices (including CGM and a mobile phone used for data collection), but all basal and bolus doses were determined and delivered according to local glycemic management guidelines without automated adjustment.

The clinical team monitored equipment operation daily. Before MRI or CT scans, the equipment was detached and reattached immediately afterward. They were removed before surgery and reactivated promptly postoperatively. Participants were asked to remain within 5 m of the controller smartphone to maintain Bluetooth connectivity. A 24 h endocrine specialist hotline provided medical support.

The study treatment was administered from enrollment until discharge, with a maximum duration of 14 days. During the study period, participants were allowed to carry out their routine daily activities, make autonomous dietary choices, and engage in indoor/outdoor physical exercise. Data from insulin pumps and CGM records were uploaded and stored on the cloud platform of United Imaging Healthcare Surgical Technology Corporation. Upon study completion, all datasets were uniformly extracted via this platform for centralized analysis.

### 2.3. Blood Glucose Data Processing

To ensure accuracy and consistency across groups, all CGM data were calibrated using fingerstick blood glucose (FBG) measurements following a standardized, validated procedure that was applied identically to both the closed-loop and control groups.

Before each meal, FBG levels were measured and paired with CGM readings within a ±30 min window, provided at least three CGM values were available in that interval. The mean difference between raw CGM values and FBG was calculated as follows:

$$\Delta =mean\left({CGM}_{raw}-FBG\right)$$

The daily average deviation (Δ) was then used to calibrate all CGM values for that day:

$${CGM}_{calibrated}=CGM_{raw}-\Delta$$

This retrospective calibration was performed to reduce systematic measurement drift and ensure consistent glycemic reporting between groups. It represents a conservative, safety-oriented adjustment that avoids artificial inflation of glucose readings and does not alter the relative comparison between treatment arms. All statistical analyses were performed using calibrated CGM data.

### 2.4. Outcomes

The primary outcome was the percentage of time in the target glucose range (3.9–10.0 mmol/L), including all available sensor-derived data during the perioperative period (defined as 2 days before surgery to 5 days after surgery).

Secondary endpoints included the mean sensor glucose concentration; the percentage of time in range (TIR) at 4.4–10.0 mmol/L; time above range (TAR), stratified as >10.0 mmol/L, >13.9 mmol/L, or >20.0 mmol/L; time below range (TBR), categorized as <3.9 mmol/L or <3.0 mmol/L; total daily insulin dose; and glycemic variability, quantified by the standard deviation (SD) and coefficient of variation (CV) of sensor glucose values throughout the study period. All primary and secondary outcomes were analyzed for the entire 24 h period, as well as for separate daytime (06:00–22:00) and nocturnal (22:00–06:00) intervals. Safety evaluation included time below range (TBR), adverse events (AEs), and device deficiencies.

### 2.5. Sample Size Calculation

The sample size calculation was performed using R version 4.2.1 (package pwr) to determine the minimum number of participants required to detect a clinically significant difference in time-in-range (TIR). Based on the previous clinical data of the general surgery department of our hospital, the baseline TIR of CGM patients receiving conventional insulin pump treatment was 47%. Assuming a two-sided type I error (α) of 0.05, a statistical power of 80% (β = 0.20), an expected absolute TIR difference of 20% between the hybrid closed-loop (HCL) and control groups, and a standard deviation of 20%, the minimum sample size was calculated as 22 participants per group. Accounting for a projected 20% attrition rate due to intervention discontinuation or loss to follow-up, the final enrollment target was set at 27 participants per group.

### 2.6. Statistical Analysis

Statistical procedures strictly adhered to the intention-to-treat (ITT) principle. Specifically, 4 participants who discontinued the study due to device-related issues were retained in the ITT analysis. The final ITT population included all 49 randomized participants (25 in the closed-loop group and 24 in the control group). No missing data imputation was performed; only available data up to the time of discontinuation were included in the analysis. Outcome measures were computed using Python 3.11 (Python Software Foundation), while hypothesis testing and data modeling were implemented in SPSS Statistics version 29 (IBM Corp.). Normally distributed continuous variables were compared using unpaired Welch’s *t*-tests, and non-normally distributed variables were analyzed with Mann–Whitney U tests. After normality testing (e.g., Shapiro–Wilk test, *p* > 0.05), continuous data were expressed as mean ± SD; otherwise, median (Q1–Q3) was reported. A 95% confidence interval (CI) was used to describe the difference between the interventions. Categorical variables (device/safety events) were evaluated using Fisher’s exact test. All calculated *p*-values were from two-tailed tests, and *p* < 0.05 was considered statistically significant. To control for potential confounding factors, a post hoc exploratory analysis was performed. A multiple linear regression model was employed, in which baseline ALT, eGFR, glycated hemoglobin (HbA1c), and surgical grade were included as covariates when analyzing the primary outcome (TIR). The *p*-values for these secondary outcomes should be interpreted with caution as hypothesis-generating rather than confirmatory evidence.

## 3. Results

### 3.1. Participant Characteristics

From October 2023 to January 2025, a total of 62 potential participants were screened for this study. Of these, 10 were excluded based on the pre-defined inclusion and exclusion criteria, and 3 withdrew their informed consent prior to randomization. Consequently, 49 participants were successfully randomized, with 25 allocated to the closed-loop group and 24 to the control group. Three participants in the closed-loop group and one in the control group discontinued the study due to device-related issues, and none of them completed the protocol-required 72 h of valid CGM monitoring. All randomized participants were included in the intention-to-treat (ITT) population for the primary efficacy analysis.

Baseline characteristics are presented in Table 1. Participants in the two groups underwent abdominal (closed-loop vs. control, 36% vs. 42%), orthopedic (28% vs. 29%), thoracic (24% vs. 25%), or neurosurgery (12% vs. 4%), with comparable demographics except for slightly lower eGFR (*p* = 0.028) and higher alanine aminotransferase (ALT) levels (*p* = 0.033) in the closed-loop group.

### 3.2. Efficacy Outcomes

The research findings are presented in detail in Table 2. Compared with the control group, the closed-loop group achieved a significantly higher time in range (TIR; 3.9–10.0 mmol/L) during hospitalization (76.4 ± 14.1% vs. 61.2 ± 20.0%, *p* = 0.005).

**Figure 2:**
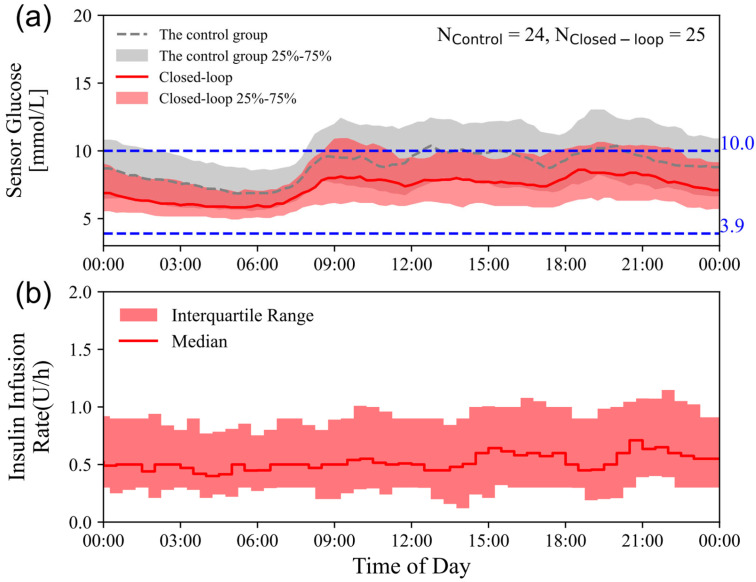
(**a**) The median sensor glucose concentrations (interquartile ranges, IQRs) are illustrated for both the closed-loop (red line and shaded area; n = 25) and control (dashed line and shaded area; n = 24) groups. The blue lines represent the target glucose range (3.9–10.0 mmol/L). (**b**) Algorithm-guided insulin infusion during the closed-loop period. The red line represents the median, with the shaded area indicating the IQR.

**Figure 3:**
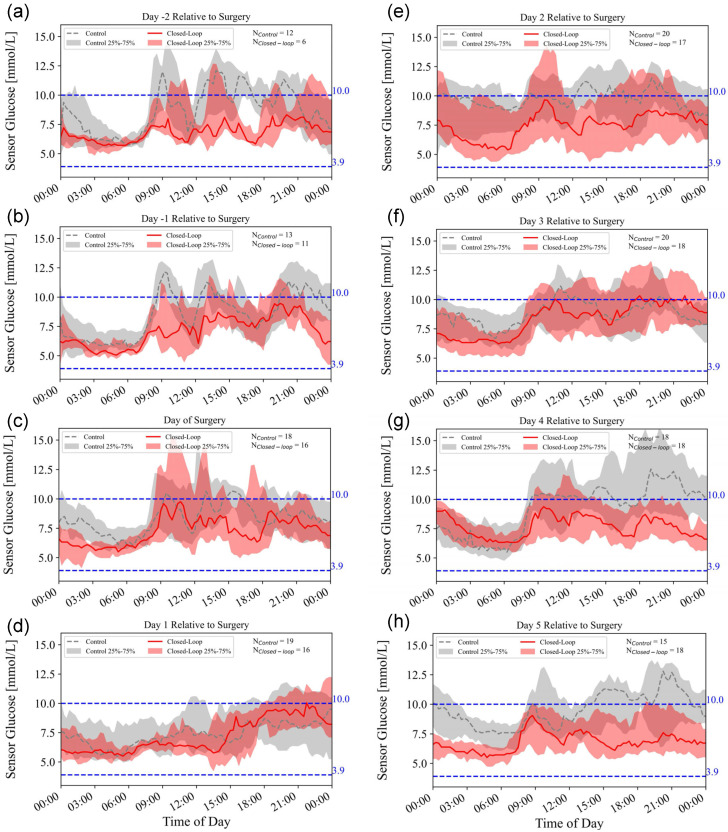
Glycemic profiles of the two groups on different surgical days, with time points labeled relative to the day of surgery: (**a**) 2 days preoperatively; (**b**) 1 day preoperatively; (**c**) day of surgery; (**d**) 1st day postoperatively; (**e**) 2nd day postoperatively; (**f**) 3rd day postoperatively; (**g**) 4th day postoperatively; (**h**) 5th day postoperatively.

The medians (interquartile ranges) of sensor glucose concentrations over time for both groups are illustrated in Figure 2a, and glycemic profiles on different surgical days between the two groups are shown in Figure 3.

For secondary glycemic endpoints, the closed-loop system demonstrated significant enhancements across multiple glucose metrics. Specifically, it achieved a 13.8 percentage point increase in the time spent within the 4.4–10.0 mmol/L range (*p* = 0.008), an 18.5 percentage point higher proportion of time within the tight glycemic range of 3.9–7.8 mmol/L (*p* < 0.001), and a 15.6 percentage point reduction in time above range (TAR; >10.0 mmol/L) (*p* = 0.004). There were no significant differences in the proportions of time >13.9 mmol/L (*p* = 0.273) or >20.0 mmol/L (*p* = 0.382). The mean glucose levels were significantly lower in the closed-loop group (8.1 ± 1.2 mmol/L) than in the control group (9.3 ± 1.7 mmol/L), with a mean difference of −1.2 mmol/L [95% CI: (−2.1 to −0.2), *p* = 0.009]. The coefficient of variation (CV) for sensor glucose showed no significant difference between the two groups. The standard deviation (SD) of the mean blood glucose level was comparable between the groups (*p* = 0.749). Time in hypoglycemia (<3.9 mmol/L and <3.0 mmol/L) and the total daily insulin dose showed no statistically significant differences between groups (all *p* > 0.05). Figure 2b presents the algorithm-guided insulin infusion during the closed-loop period.

We also compared the efficacy of glucose control between the two groups in different time periods (daytime and nighttime), the results of which are shown in detail in Table 3. The closed-loop group exhibited a significantly higher TIR (3.9–10.0 mmol/L) during both daytime (*p* = 0.002) and nighttime (*p* = 0.047), a significantly lower TAR (>10 mmol/L) during both daytime (*p* = 0.002) and nighttime (*p* = 0.022), and a significantly lower average sensor glucose level throughout all time periods (all *p* < 0.05). During the daytime, the closed-loop group demonstrated a 16.7 percentage point increase in TIR (if defined as 4.4–10.0 mmol/L) compared to the control group (*p* = 0.003), while the nighttime difference was not significant (*p* = 0.112). No significant between-group differences were observed in glycemic variability indices (CV, SD), time above range (TAR; >13.9 or >20.0 mmol/L), and time below range (TBR; <3.9 or <3.0 mmol/L) during either daytime or nighttime periods (all *p* > 0.05).

To control for potential confounding effects of baseline imbalances and surgical factors, we performed a multivariable linear regression analysis. After adjusting for surgery type, surgical grade, baseline ALT, baseline eGFR, and baseline HbA1c, the treatment allocation (hybrid closed-loop system vs. conventional insulin pump) remained an independent predictor of TIR (adjusted β = 27.49%, 95% CI: 5.33% to 49.65%; *p* = 0.019). In contrast, baseline ALT, eGFR, HbA1c, and surgery-related variables were not significantly associated with TIR (all *p* > 0.05).

### 3.3. Safety Outcomes

With regard to adverse glycemic events, no severe hypoglycemic episodes (blood glucose < 2.2 mmol/L) were detected in either group. Five cases of hyperglycemia (blood glucose > 20 mmol/L) were recorded in both the closed-loop group and the control group. Critically, neither group observed any episodes of ketosis (blood ketones > 1.0 mmol/L), indicating effective metabolic control. Additionally, no serious device-related adverse events were reported in either group. However, it should be noted that this pilot study was underpowered to detect rare but clinically critical safety events, such as severe hypoglycemia or ketoacidosis. Safety outcomes are summarized in Table 4.

## 4. Discussion

This is a randomized trial to evaluate the efficacy and safety of an open-source hybrid closed-loop AID system in perioperative patients with diabetes (excluding type 1 diabetes) in China. Our findings demonstrate that, compared with conventional insulin pump therapy combined with CGM, the open-source AID system significantly improved glycemic control without increasing the risk of hypoglycemia during the perioperative period.

The most notable finding of this trial was a significant improvement in TIR (3.9–10.0 mmol/L) in the closed-loop group (open-source AID system) compared with the control group (conventional insulin pump) (76.4 ± 14.1% vs. 61.2 ± 20.0%, *p* = 0.005), representing a 15.2 percentage point improvement that was achieved without increasing hypoglycemic risk (TBR). This result substantiates the efficacy of automated insulin delivery in the complex perioperative setting, a finding consistent with the earlier study by Herzig et al., which confirmed that fully closed-loop insulin delivery is superior to standard insulin therapy in glucose control in perioperative patients [13]. Furthermore, the magnitude of TIR improvement observed in our study aligns with the robust effects demonstrated by commercial closed-loop systems in other high-risk inpatient populations. For instance, in a landmark trial by Thabit et al. involving general ward inpatients with type 2 diabetes, a commercial closed-loop system increased TIR (5.6–10.0 mmol/L) by 21.8 percentage points [23]. Similarly, in a more complex cohort of inpatients receiving enteral or parenteral nutrition, Boughton et al. reported a 32.0 percentage point increase in TIR (5.6–10.0 mmol/L) with fully closed-loop delivery [24].

The superiority of the AID system was evident across both daytime and nighttime periods. Nighttime glycemic control is particularly challenging in inpatient settings due to reduced nursing surveillance and variable insulin sensitivity. Our study showed that the closed-loop group maintained a significantly higher TIR and lower TAR during the night, and the mean glucose was also lower in the closed-loop group during the night, highlighting the benefit of automated insulin adjustments in maintaining stable overnight glucose levels. This is consistent with findings from studies in type 1 diabetes (T1D) populations, where open-source AID systems have shown remarkable efficacy in nocturnal glucose management [21].

One of the key strengths of this study is the successful application of an open-source AID algorithm (OpenAPS Oref0). Compared with commercial closed-loop systems, the open-source AID algorithm offers superior algorithmic transparency, customizability, and cost-effectiveness, enabling better adaptation to local clinical practices and resource-limited settings. In this study, the system was successfully deployed in a real-world clinical environment with minimal additional training for nursing staff, suggesting its feasibility for broader adoption in resource-limited settings. Optimal glycemic control achieved via this approach is critically important in the perioperative period, as hyperglycemia is an established risk factor for postoperative complications, consistent with our previous findings [10,11].

The open-source AID system demonstrated a reassuring safety profile in this trial, with no severe hypoglycemia or ketoacidosis events and comparable device-related outcomes between groups. These findings align with previous reports that open-source AID systems are generally safe when properly implemented and monitored [18,21]. While these results are encouraging, the relatively small sample size may limit the power to detect rare adverse events. Therefore, larger studies are needed to definitively confirm the long-term safety of this system in the inpatient setting.

Despite promising results, this study has limitations. First, its single-center design and relatively small sample size may limit the generalizability of the findings and reduce the statistical power to detect differences in infrequent safety endpoints, such as hypoglycemia. Larger, multicenter trials are needed to confirm the efficacy and safety of open-source AID in diverse perioperative populations. Second, our study did not account for the potential modulating effect of different surgical procedures on metabolic stress and glycemic outcomes. Notably, varying degrees of surgical stress and intraoperative physiological changes across different operation types may independently influence perioperative glucose fluctuations and control efficacy. This limitation precludes generalized conclusions across all surgery types and should be addressed in future research through procedure-specific subgroup analyses. Third, the study duration was short (generally 3–5 days), so the long-term benefits and durability of the system remain unclear. Fourth, blinding of participants and staff was not feasible due to the visible devices, introducing a risk of performance bias. Future studies should consider blinded outcome assessment by independent personnel. Finally, the system required manual input for meal boluses, which means it is not fully closed-loop. However, this hybrid approach is more practical in inpatient settings where meal timing and content are often unpredictable.

## 5. Conclusions

In conclusion, the current study demonstrated that open-source hybrid closed-loop AID systems can provide better glycemic control than conventional insulin pump therapy in perioperative patients with T2DM. These initial findings indicate that the system may represent a safe and effective option, which could be considered an alternative to conventional perioperative insulin therapy. With further validation and scalability, open-source AID may serve as a foundational approach to personalized diabetes care in surgical patients, particularly in low-resource settings where expensive commercial systems are not accessible.

## Figures and Tables

**Table 1:** Baseline characteristics.

Item	Closed-Loop (n = 25)	Control (n = 24)	*p*-Value
Sex, M/F, n	17/8	17/7	1.000
Age, yr	62.0 (57.0, 68.0)	59.0 (53.5, 62.5)	0.268
BMI, kg/$m^{2}$	23.2 ± 3.2	24.2 ± 2.5	0.250
BW, kg	64.3 ± 10.4	69.9 ± 11.2	0.110
${HbA}_{1C}$, %	8.64 ± 1.66	8.91 ± 0.93	0.621
FBG	9.0 ± 3.0	10.2 ± 4.1	0.303
Duration of diabetes, yr	10 (0.63,20)	10 (8,16)	0.549
Newly diagnosed diabetes, n	3	1	0.609
Glucose-lowering treatment at admission			
Insulin therapy, n	5	5	1.000
Metformin, n	7	8	0.924
Sulfonylurea, n	4	2	0.667
GLP-1 therapy, n	0	1	0.490
Glucosidase inhibitor, n	3	5	0.653
DPP-IV inhibitor, n	1	0	1.000
SGLT-2 inhibitor, n	1	2	0.971
Charlson comorbidity index	7.12 ± 2.71	7.05 ± 2.25	0.41
Nutritional therapy			1.000
Yes	15	15	
No	10	9	
Surgical grade			
Grade III	5	2	0.417
Grade IV	20	22	
Surgical classification, n (%)			
Abdominal surgery	9 (36%)	10 (42%)	0.909
Neurosurgery	3 (12%)	1 (4%)	0.609
Thoracic surgery	6 (24%)	6 (25%)	1.000
Orthopedic surgery	7 (28%)	7 (29%)	1.000
Cr, μmol/L	83.7 ± 51.8	64.0 ± 18.7	0.114
eGFR, mL/min/1.73 m^2^	86.9 ± 24.7	98.5 ± 11.8	0.028
TC, mmol/L	4.09 ± 1.00	4.45 ± 1.33	0.420
TGs, mmol/L	1.11 (0.79, 2.67)	1.81 (0.57, 2.88)	0.887
ALT, U/L	21 (11, 45)	15 (9, 17)	0.033
AST, U/L	21 (16, 52)	20 (15, 25)	0.235

Data are presented as mean ± SD for normally distributed variables and median (IQR) for non-normally distributed variables. SD, standard deviation; IQR, interquartile range; M, male; F, female; BW, body weight; yr, years; FBG, fasting blood glucose; GLP-1, glucagon-like peptide-1; DPP-IV, dipeptidyl peptidase-IV; SGLT-2, sodium-dependent glucose transporters 2; Cr, creatinine; BMI, body mass index; eGFR, estimated glomerular filtration rate; TC, total cholesterol; TGs, triglycerides; ALT, alanine aminotransferase; AST, aspartate aminotransferase.

**Table 2:** Comparison of 24 h perioperative glycemic outcomes between the closed-loop and control groups.

Outcome	Closed-Loop (n = 25)	Control (n = 24)	Group Difference	95% CI	*p*-Value
TIR with glucose 3.9–10.0 mmol/L, %	76.4 ± 14.1	61.2 ± 20.0	15.2	[4.9, 25.4]	0.005
Mean glucose, mmol/L	8.1 ± 1.2	9.3 ± 1.7	−1.2	[−2.1, −0.2]	0.009
SD of glucose, mmol/L	2.7 ± 1.0	2.7 ± 0.7	−0.0	[−0.7, 0.4]	0.749
CV of glucose, %	33.0 ± 8.3	29.8 ± 7.7	3.2	[−2.6, 8.7]	0.267
Between-days CV of glucose, %	16.0 ± 5.8	16.4 ± 7.6	−0.4	[−5.3, 5.0]	0.617
TIR with glucose 4.4–10.0 mmol/L, %	73.6 ± 14.0	59.8 ± 19.7	13.9	[3.8, 24.0]	0.008
TIR with glucose 5.6–10.0 mmol/L, %	59.5 ± 12.9	52.0 ± 17.0	7.5	[−1.4, 16.4]	0.096
TIR with glucose 3.9–7.8 mmol/L, %	53.6 ± 16.1	35.1 ± 17.9	18.5	[8.5, 28.5]	<0.001
TAR with glucose > 10.0 mmol/L, %	21.4 ± 14.2	37.1 ± 20.5	−15.6	[−26.0, −5.2]	0.004
TAR with glucose > 13.9 mmol/L, %	3.4 (0.2, 7.6)	6.9 (1.6, 13.2)	−4.4	[−9.6, 1.2]	0.273
TAR with glucose > 20.0 mmol/L, %	0.0 (0.0, 0.0)	0.0 (0.0, 0.0)	0.3	[0.0, 0.0]	0.382
TBR with glucose < 3.9 mmol/L, %	1.2 (0.7, 2.5)	0.7 (0.0, 1.7)	0.4	[−0.3, 1.8]	0.102
TBR with glucose < 3.0 mmol/L, %	0.0 (0.0, 0.0)	0.0 (0.0, 0.1)	−0.0	[−0.1, 0.0]	0.205
Total daily insulin dose, U/kg/day	0.36 ± 0.17	0.47 ± 0.16	−0.08	[−0.2, 0.05]	0.204

Data are presented as mean ± SD or median (IQR). TIR, time in range; TAR, time above range; TBR, time below range; CV, coefficient of variation. Glycemic outcomes were derived from the SIBIONICS^®^ continuous glucose monitoring system during hospitalization.

**Table 3:** Daytime and nighttime glycemic control during the perioperative period.

Outcome	Closed-Loop (n = 22)	Control (n = 23)	Group Difference	95% CI	*p*-Value
**Nighttime period from 22:00 to 06:00**					
TIR with glucose 3.9–10.0 mmol/L, %	88.5 (80.7, 98.0)	76.6 (68.5, 92.4)	11.9	[−1.7, 23.1]	0.047
TIR with glucose 4.4–10.0 mmol/L, %	82.6 (73.1, 96.4)	73.8 (66.2, 89.0)	8.8	[−1.2, 20.0]	0.112
Mean glucose, mmol/L	6.6 (5.8, 7.8)	7.9 (6.9, 8.9)	−1.4	[−2.1, −0.2]	0.004
SD of glucose, mmol/L	1.8 ± 0.9	2.1 ± 0.7	−0.3	[−0.8, 0.1]	0.160
CV of glucose, %	25.3 ± 9.2	26.2 ± 7.5	−0.8	[−5.7, 4.1]	0.742
Between-nighttime CV of glucose, %	19.9 ± 8.3	22.2 ± 8.1	−2.3	[−7.1, 2.5]	0.348
TAR with glucose > 10.0 mmol/L, %	2.1 (0.0, 13.5)	20.9 (2.9, 30.3)	−18.8	[−26.5, −1.8]	0.022
TAR with glucose > 13.9 mmol/L, %	0.0 (0.0, 1.2)	0.0 (0.0, 3.5)	0.0	[−2.8, 0.0]	0.279
TAR with glucose > 20.0 mmol/L, %	0.0 (0.0, 0.0)	0.0 (0.0, 0.0)	0.0	[0.0, 0.0]	1.000
TBR with glucose < 3.9 mmol/L, %	0.2 (0.0, 4.2)	0.1 (0.0, 2.8)	0.1	[−1.6, 2.8]	0.686
TBR with glucose < 3.0 mmol/L, %	0.0 (0.0, 0.0)	0.0 (0.0, 0.0)	0.0	[0.0, 0.0]	0.793
**Daytime period from 06:00 to 22:00**					
TIR with glucose 3.9–10.0 mmol/L, %	73.2 ± 16.0	55.3 ± 21.2	17.8	[6.7, 28.9]	0.002
TIR with glucose 4.4–10.0 mmol/L, %	70.9 ± 15.7	54.2 ± 20.7	16.7	[5.9, 27.6]	0.003
Mean glucose, mmol/L	8.2 (7.5, 9.0)	9.5 (8.5, 10.5)	−1.3	[−2.2, −0.5]	0.007
SD of glucose, mmol/L	2.7 (2.0, 3.2)	2.7 (2.3, 3.2)	−0.0	[−0.6, 0.4]	0.779
CV of glucose, %	31.8 ± 7.1	28.7 ± 8.0	3.1	[−1.4, 7.5]	0.174
Between-daytime CV of glucose, %	16.7 (12.4, 25.5)	14.9 (11.2, 20.2)	1.8	[−3.3, 10.5]	0.219
TAR with glucose > 10.0 mmol/L, %	24.9 ± 16.2	43.1 ± 21.8	−18.1	[−29.5, −6.8]	0.002
TAR with glucose > 13.9 mmol/L, %	3.7 (0.2, 7.8)	8.6 (2.3, 16.7)	−4.9	[−12.4, 1.0]	0.191
TAR with glucose > 20.0 mmol/L, %	0.0 (0.0, 0.0)	0.0 (0.0, 0.0)	0.0	[0.0, 0.0]	0.390
TBR with glucose < 3.9 mmol/L, %	1.2 (0.7, 2.7)	0.5 (0.0, 1.8)	0.7	[−0.2, 2.2]	0.133
TBR with glucose < 3.0 mmol/L, %	0.0 (0.0, 0.0)	0.0 (0.0, 0.1)	−0.0	[−0.1, 0.0]	0.112

Data are presented as mean ± SD or median (IQR). TIR, time in range; TAR, time above range; TBR, time below range; CV, coefficient of variation. Glycemic outcomes were derived from the SIBIONICS^®^ continuous glucose monitoring system during hospitalization.

**Table 4:** Safety outcomes and device issues.

Events (No.)	Closed-Loop Group	Control Group
Hyperglycemic event (blood glucose > 20 mmol/L)	5	5
Severe hyperglycemic events *	0	0
Severe hypoglycemic events (blood glucose < 2.2 mmol/L)	0	0
CGM data loss	5	2
Catheter occlusion	1	0
Serious device adverse events	0	0

No. is the number of events; “*” denotes severe hyperglycemia (blood glucose > 20 mmol/L) with concurrent ketonemia (β-hydroxybutyrate > 1 mmol/L).

## Data Availability

The original contributions presented in this study are included in the article. Further inquiries can be directed to the corresponding author.

## Abbreviations

The following abbreviations are used in this manuscript:

T2DM	type 2 diabetes mellitus
T1DM	type 1 diabetes mellitus
AID	automated insulin delivery
APS	artificial pancreas systems
CGM	continuous glucose monitor
CSII	continuous subcutaneous insulin infusion
ALT	alanine Aminotransferase
AST	aspartate aminotransferase
HCL	hybrid closed loop
OREF0	Open APS Reference Design Zero
TIR	percentage of time in range
TAR	time above range
TBR	time below range
SD	standard deviation
CV	coefficient of variation
IQR	interquartile range
SAEs	serious adverse events
